# Development and validation of a non-invasive prediction model for identifying high-risk children with metabolic dysfunction-associated fatty liver disease

**DOI:** 10.3389/fped.2025.1625864

**Published:** 2025-08-06

**Authors:** Yuan Xiaowu, Dong Jian, Wen Yizhu, Ma Ting, Tong Jin, Wei Juan, Wang Sirui, Wang Jinli, He Yuchen, Zhao Huan, Cheng Yuhang, Li Jun

**Affiliations:** Department of Ultrasound, The First Affiliated Hospital, Shihezi University, Shihezi, China

**Keywords:** metabolic dysfunction-associated fatty liver disease, non-alcoholic fatty liver disease, pediatric population, risk stratification, health index

## Abstract

**Objective:**

This study aims to investigate the prevalence and risk factors of Metabolic dysfunction-associated fatty liver disease (MAFLD) in pediatric populations and establish a novel health index scoring system derived from key risk parameters for early identification of high-risk children with MAFLD.

**Method:**

In this cross-sectional study, a systematic random sampling method was employed to recruit children (6–18 years) with MAFLD. Data collection involved standardized questionnaires and comprehensive anthropometric measurements. The prevalence of MAFLD was determined through epidemiological analysis. Both univariate and multivariate logistic regression models were systematically applied to identify independent risk factors (*P* *<* 0.05), with subsequent development of a health index scoring system. The optimal diagnostic threshold for the health index was established using receiver operating characteristic (ROC) curve analysis.

**Results:**

The study cohort comprised 2,190 pediatric participants, revealing an overall MAFLD prevalence of 26.30%. Significant demographic disparities were observed: males exhibited a higher prevalence than females. The age, BMI (Body Mass Index), Waist-Hip Ratio (WHR), and Waist-Height Ratio (WHtR) values of the MAFLD group were higher than those of the Non-MAFLD group, and the difference was statistically significant. Multivariable logistic regression subsequently identified seven independent predictors (*P* < 0.05), age (OR = 1.62, 95% CI 1.36, 1.92), gender (OR = 0.42, 95% CI 0.31,0.57), BMI (OR = 2.15, 95% CI 1.75, 2.64), WHR (OR = 2.10, 95% CI 1.64, 2.69), WHtR (OR = 4.01, 95% CI 3.07, 5.23), sleep duration (OR = 0.71, 95% CI 0.59, 0.85) and dessert consumption (OR = 1.46, 95% CI 1.17, 1.81). Health index demonstrated moderate predictive accuracy in both training (AUC = 0.72, 95% CI 0.68, 0.76) and validation cohorts (AUC = 0.74, 95% CI 0.70, 0.78) with optimal diagnostic threshold at 11.5 points. Calibration analysis revealed satisfactory model fit (Hosmer-Lemeshow *χ*^2^ = 7.32, *P* = 0.12). Strong concordance was observed between dimension weights and regression coefficients (Pearson's *r* = 0.93, *P* < 0.001).

**Conclusion:**

This study establishes seven independent determinants of MAFLD in pediatric populations: age, gender, BMI, waist-hip ratio, waist-height ratio, sleep duration, and frequent dessert consumption (*P* *<* 0.05). The health index demonstrates robust clinical utility for early detection, providing an evidence-based screening protocol for school health programs. Implementation of this quantitative tool could significantly enhance targeted prevention strategies and optimize resource allocation in childhood metabolic disorder surveillance in communities.

## Background

Metabolic dysfunction-associated fatty liver disease (MAFLD), introduced in 2020 by a multinational panel of experts through the International Consensus Statement, represents a paradigm shift from the previous diagnostic label of Non-alcoholic Fatty Liver Disease (NAFLD) (1). Similar to NAFLD, MAFLD is characterized by excessive hepatocellular lipid deposition (≥5%) as its pathognomonic histopathological feature (2). The disease spectrum encompasses simple steatosis, Metabolic Associated Steatohepatitis (MASH), progressive hepatic fibrosis, and hepatocellular carcinoma (HCC) (3). Emerging as the leading etiology of pediatric chronic liver disease globally, MAFLD demonstrates a multisystem metabolic perturbation profile that transcends hepatic pathology. Contemporary epidemiological data reveal its pathophysiological continuum with type 2 diabetes mellitus (T2DM), cardiovascular diseases (CVD), metabolic syndrome (MetS), and insulin resistance (4). Epidemiological surveillance data document a striking 2.7-fold surge in pediatric MAFLD prevalence rates worldwide, escalating from 3.9% in 2000 to 10.6% by 2020. Particularly alarming is the 34.2% prevalence observed in obese pediatric cohorts, establishing childhood obesity as the cardinal modifiable risk determinant. Geospatial analysis reveals accelerated incidence trajectories in Asian regions, with China and India demonstrating epidemiological transition patterns strongly correlated with Westernized dietary patterns and pervasive sedentarism (5–8). The predictive modeling framework developed by Li, J. et al. projects a staggering escalation in pediatric MAFLD prevalence, with global estimates reaching 30.7% by 2040, Asian populations demonstrate the most alarming trajectory, peaking at 49.7% (9). This epidemic acceleration correlates strongly with sustained obesogenic environments and epigenetic reprogramming induced by developmental overnutrition (9).

Compared with adults, MAFLD in children has unique clinical and histologic features due to its unique physiological stage. (1) Rapidly progressive: about 9.3% of children progressed to MASH within 5 years, significantly higher than adults (3.6%) (10). (2) Metabolic dysfunction is prominent, with more than 80% of children having comorbid insulin resistance (11). (3) Genetic predisposition is significant. The transmission rate of PNPLA3 rs738409 allele (GG type) was 1.5 times higher in children than in adults, and it was associated with early-onset severe fatty liver disease (12). It is more likely to cause serious intrahepatic and extrahepatic adverse outcomes, such as liver cirrhosis, HCC, and cardiovascular-related diseases (13, 14). However, typical symptoms are often absent in pediatric patients with NAFLD, but MAFLD-induced cognitive decline, low self-esteem, and social impairment have burdened children.

Early screening can detect MAFLD in children, and then through lifestyle intervention, 30% of liver fat can be reversed (15). However, mass screening may trigger unnecessary family anxiety or excessive medical behavior, and at the same time cause a waste of medical resources (16). Therefore, the identification of high-risk children can not only detect children with MAFLD in time, but also reduce the anxiety of patients' families and reduce the waste of medical resources.

In this study, we innovatively constructed a multidimensional health index scoring system, integrating demographic characteristics, anthropometric indicators, and behavioral parameters (17). Predictive models offer more comprehensive, accurate, and cost-effective disease prevention than single indicators (17–19). This study aimed to identify high-risk groups for MAFLD in children and provide a basis for accurate screening of MAFLD.

## Methods

From January 2024 to December 2024, a cross-sectional survey was conducted at a middle school in Shihezi City, Xinjiang Uyghur Autonomous Region, China. A total of 2,280 students aged 6–18 years were included in the systematic cluster sampling method for routine ultrasound screening. According to Guidelines for the prevention and treatment of metabolic dysfunction-associated (non-alcoholic) fatty liver disease (Version 2024) diagnostic criteria (20), combined with questionnaire survey and clinical data, 2,190 cases were finally included in the study cohort after strict screening criteria. Inclusion Criteria: (1) Ultrasonography meets diagnostic criteria for MAFLD; (2) Age 6–18 years old; (3) Written informed consent signed by study subjects and their legal guardians; (4) Complete the baseline questionnaire and clinical examination items. Exclusion Criteria: (1) Patients with viral hepatitis, drug-induced liver injury, hereditary metabolic liver disease, type I diabetes mellitus and other chronic liver and kidney diseases; (2) Those who have had more than 3 ultrasound examinations but still have poor image quality and cannot be diagnosed; (3) Key clinical data or questionnaire items are missing >10%.

In this study, a cross-sectional study design was adopted, and data collection was carried out based on the Assessment Scale of Risk Factors for Metabolism-related Fatty Liver Disease in Children made by our research group. After being reviewed by the Ethics Committee of the First Affiliated Hospital of Shihezi University (approval number: KJ2024-455-02), 2,280 school-age children aged 6–18 years were included by cluster sampling. The reliability and validity of the questionnaire were verified by pre-experiments, and the Cronbach's *α* coefficient was 0.82, and the content validity index was 0.90.

### Data collection

The uniformly trained investigator will explain the purpose of the study to the legal guardian of the subject in detail and sign the informed consent form, and complete the questionnaire for the subject with comprehension impairment with the assistance of the legal guardian and the guidance of the trained medical staff. 2,280 questionnaires were distributed, 2,230 questionnaires were recovered, the questionnaire distribution and recovery rate was 97.81%, and 2,190 valid questionnaires (effective rate 98.21%) were verified for completeness.

Existing studies have confirmed the effects of age, gender, BMI, WHR, WHtR, diet, exercise, and other factors on MAFLD. This study aims to develop a noninvasive prediction model based on these reported MAFLD-related factors (17, 21–23). Variable classification: Demographic characteristics: name, gender, age; Physical measurements: height, weight, waist circumference, hip circumference; History of metabolic diseases: hypertension, diabetes, chronic kidney disease; Lifestyle: smoking status, drinking behavior, daily sleep duration, weekly exercise duration; Dietary evaluation: staple food structure (coarse grains, fine grains), frequency of breakfast, night snack, milk, eggs, soy products, coffee and desserts per week. Data quality control: Two people independently enter data, logical verification eliminates contradictory entries, Tukey's fences method is used to identify the outliers of continuous variables, and multiple imputation methods are used for missing data processing.

### Physical examination

Anthropometric data collection strictly follows a standardized operating procedure: all subjects are fasted for ≥8 h and are completed by standardized trained measurologists using uniformly calibrated measuring equipment. (1) The upright height was measured with a mechanical height measuring instrument (accuracy 0.1 cm). (2) Body weight was determined using a calibrated electronic scale (accuracy 0.01 kg). (3) According to the WHO recommended method, the waist circumference was measured at the midpoint of the upper edge of the iliac crest and the lower edge of the costal arch using a non-elastic tape measure (accuracy of 0.1 cm), and the hip circumference was measured at the most prominent point of the greater trochanteric of the femur (each index was measured consecutively for 3 times and averaged). Based on the above basic data, the following parameters are calculated: Body Mass Index [BMI, BMI = weight (kg)/height^2^ (m^2^)], Waist-to-Hip Ratio [WHR, WHR = Waist (cm)/Hip (cm)], and Waist-to-Height Ratio [WHtR, WHtR = Waist (cm)/height (cm)]. All measurements were performed by following the International Standard for Anthropometric Methods (ISAK, 2011 Edition), and the measurements were made in such a way that the subjects were wearing only light underwear (24).

### Diagnostic criteria

Based on current guidelines and expert consensus, the diagnostic criteria for hepatic steatosis using ultrasound are detailed below. (1) grade 0, normal echogenicity; (2) grade 1, slight, diffuse increase in fine echoes in liver paren chyma with normal visualization of diaphragm and intrahe patic vessel borders; (3) grade 2, moderate, diffuse increase in fine echoes with slightly impaired visualization of intrahepatic vessels and diaphragm; (4) grade 3, marked increase in fine echoes with poor or nonvisualization of the intrahepatic vessel bor ders, diaphragm, and posterior right lobe of the liver (20, 25, 26). Two sonographers with more than 5 years of work experience screened the study subjects for fatty liver disease, and when the two doctors disagreed, a third doctor with 10 years of work experience was consulted for diagnosis.

### Indicator building

Multivariate logistic regression was used to screen potential risk variables (*α* = 0.05), and the variables with independent predictive power were determined by the backward stepwise regression method (*α* = 0.05). Based on the *β* values of the regression coefficients, a weighted comprehensive health index scoring system was constructed, and the specific scoring rules were the integer approximations of the standardized regression coefficients of each variable. The health index was used as a new predictor to evaluate the differentiation of MAFLD by the receiver operating characteristic curve (ROC), and the Youden index method was used to determine the optimal diagnostic cut-off value. The area under the curve (AUC) and the 95% confidence interval (95% CI) were calculated at the same time.

### Statistical methods

Statistical analyses were performed using the Python programming language (version 3.10.12), with additional support from the following open-source libraries: NumPy (v1.24.3), pandas (v2.0.3), SciPy (v1.10.1), and statsmodels (v0.14.0). Continuous variables were first standardized to standard deviation (Z-Score normalized), and then normality was assessed by the Shapiro–Wilk test, and those who met the normal distribution were expressed as mean ± standard deviation ($\bar{x}\pm s$), and an independent samples *t*-test was used for comparison between groups. Non-normally distributed data were presented as median (M, P25, P75), and the Mann–Whitney U test was used for comparison between groups. Categorical variables were expressed as frequencies (composition ratios), and comparisons between groups were performed using either the chi-square test or Fisher's exact test. The MAFLD-related factors were preliminarily screened by univariate binary logistic regression, and the variables of *P* < 0.05 in univariate analysis were included in the multivariate binary logistic regression model. Based on the independent risk factors screened out by multivariate analysis, the health index was constructed, the ROC curve was plotted to evaluate the health index to evaluate discriminant performance of MAFLD, and the AUC, 95% CI, and optimal diagnosis threshold were calculated. All statistical analyses were performed using a two-sided test, and the test level was set at *α* = 0.05.

## Results

### Basic information

**Figure 1:**
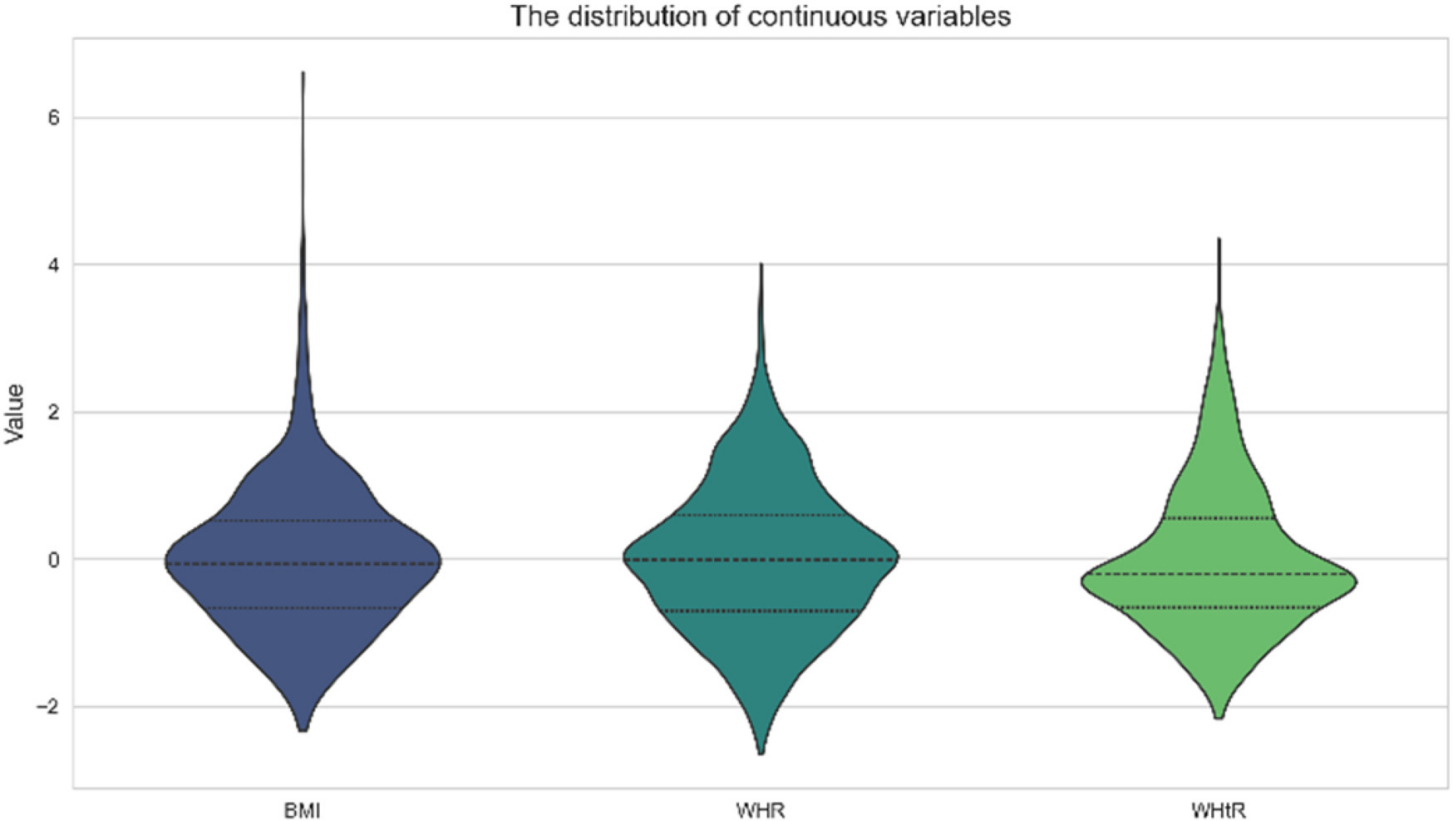
The distribution of continuous variables.

**Figure 2:**
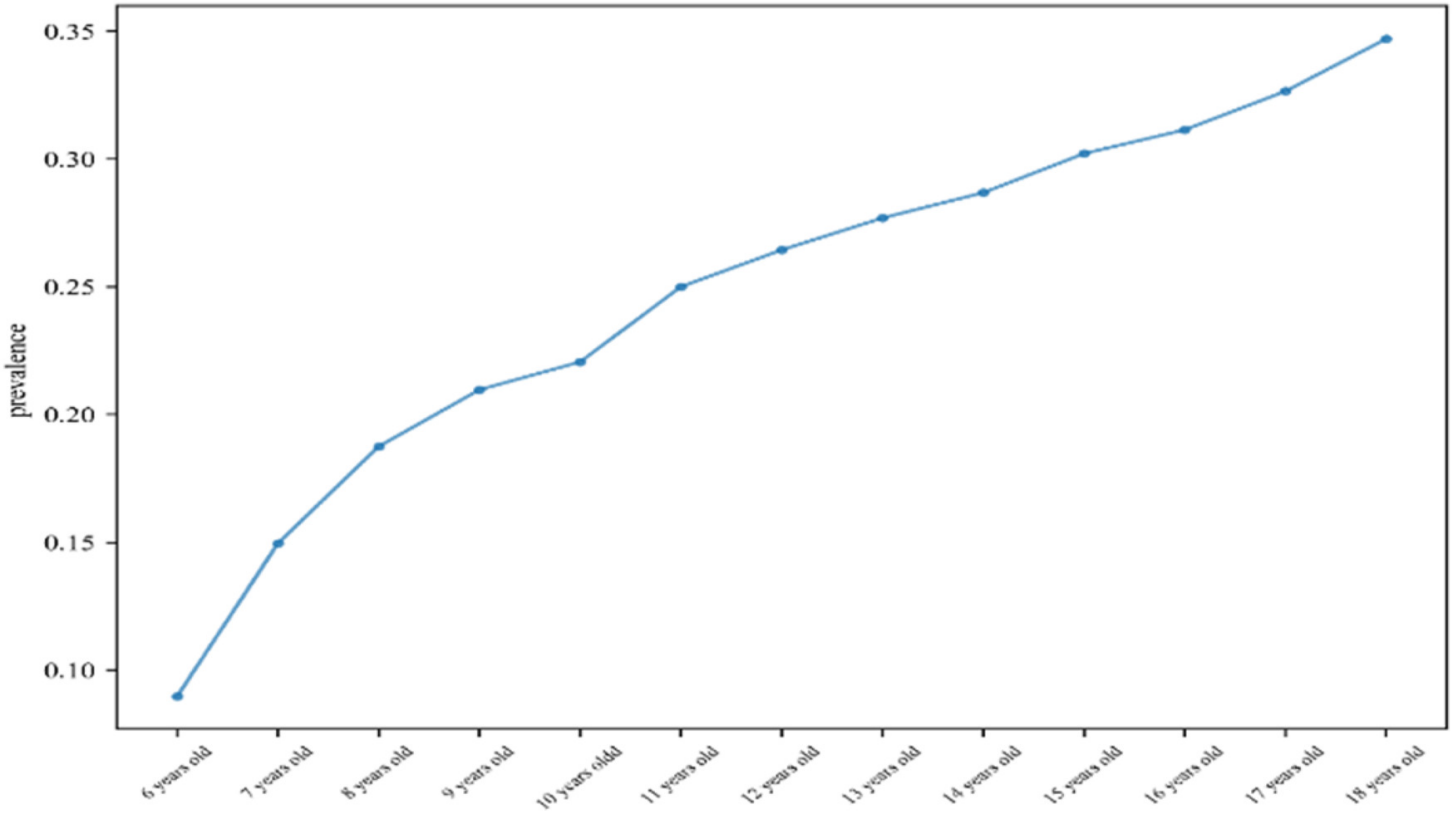
Prevalence of MAFLD in children across different age groups.

**Figure 3:**
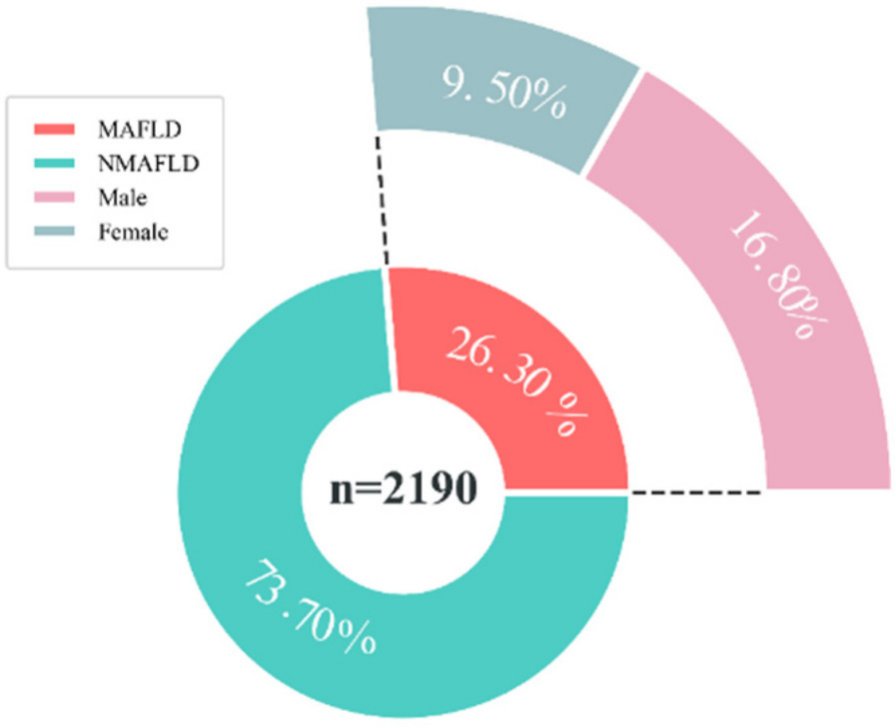
Prevalence of MAFLD in children of different genders. MAFLD, metabolic associated fatty liver disease, NMAFLD, non-metabolic associated fatty liver disease.

A total of 2,190 children were included in this study (1,024 males and 1,106 females), and the mean age, BMI, WHR, and WHtR were 12.75 ± 3.56, 19.58 ± 2.58, 0.85 ± 0.08, and 0.46 ± 0.06, respectively (Figure 1). All baseline data are presented in Table 1. Data analysis showed that there were significant differences in the detection rate of MAFLD among different age groups, and showed an increasing trend with age (Figure 2). In terms of gender distribution, there were 1,024 males (46.76%) and 1,166 females (53.24%), and there was a significant gender difference in the detection rate of MAFLD (16.80% males and 9.50% females (Figure 3).

**Table 1:** Demographic and clinical characteristics.

Characteristic	Total population	N-MAFLD	MAFLD	*χ*^2^/*t*	*P*
All	2,190	1,614	576		
Gender				10.12	<0.001
Male	1,024	656	368 (35.94%)		
Female	1,166	958	208 (17.84%)		
Age (years)	12.75 ± 3.56	12.65 ± 3.41	13.59 ± 3.29	118.39	<0.001
6	78	71	7 (8.97%)		
7	127	108	19 (14.96%)		
8	176	143	33 (18.75%)		
9	124	98	26 (20.97%)		
10	136	106	30 (22.06%)		
11	148	111	37 (25.00%)		
12	174	128	46 (26.44%)		
13	195	141	54 (27.69%)		
14	258	184	74 (28.68%)		
15	192	134	58 (30.21%)		
16	167	115	52 (31.14%)		
17	196	132	64 (32.65%)		
18	219	143	76 (34.70%)		
BMI	19.58 ± 2.58	18.90 ± 2.07	21.50 ± 2.88	−42.98	<0.001
WHR	0.85 ± 0.08	0.82 ± 0.07	0.92 ± 0.07	−38.27	<0.001
WHtR	0.46 ± 0.06	0.44 ± 0.06	0.53 ± 0.06	−19.21	<0.001
Average sleep duration (h/d)				44.76	<0.001
<6	261	207	54		
6–6.99	407	293	114		
7–7.99	840	561	279		
≥8	682	553	129		
Exercise duration (h/w)				85.97	<0.001
<1	279	219	60		
1–2.99	565	375	190		
3–4.99	863	592	271		
≥5	483	428	55		
Frequency of breakfast per week				134.38	<0.001
<1	328	260	68		
1–3	501	325	176		
4–6	637	398	239		
7	724	631	93		
Frequency of late-night snacks per week				144.04	<0.001
<1	704	631	73		
1–3	756	501	255		
4–6	523	330	193		
7	207	152	55		
Staple food composition				7.32	0.007
Coarse grains	1,266	905	361		
Fine grains	924	709	215		
Frequency of milk consumption per week				289.61	<0.001
<1	369	294	75		
1–3	356	184	172		
4–6	547	314	233		
7	918	822	96		
Frequency of egg consumption per week				455.74	<0.001
<1	273	212	61		
1–3	179	33	146		
4–6	737	470	267		
7	1,001	899	102		
Frequency of soy product consumption per week				216.76	<0.001
<1	350	278	72		
1–3	631	428	203		
4–6	487	260	227		
7	722	648	74		
Frequency of coffee consumption per week				291.62	<0.001
<1	227	171	56		
1–3	345	183	162		
4–6	570	324	246		
7	1,048	936	112		
Dessert consumption frequency per week				150.52	<0.001
<1	611	502	109		
1–3	1,084	852	232		
4–6	429	226	203		
7	66	34	32		

N-MAFLD, non-metabolic associated fatty liver disease; MAFLD, metabolic associated fatty liver disease; BMI, body mass index; WHR, waist-to-hip ratio; WHtR, waist-to-height ratio; h/d, hour/day; h/w, hour/week.

### Univariate analysis

In this study, MAFLD was used as the outcome measure, and the variables were initially screened by univariate logistic regression analysis (*α* = 0.05). The *P* values of the five variables of smoking *(P* = 0.12), alcohol consumption (*P* = 0.86), hypertension (*P* = 0.62), diabetes mellitus (*P* = 0.78) and kidney disease (*P* = 0.38) were all >0.05, and the fifteen variables were finally included in age, gender, BMI, WHR, WHtR, sleep, exercise, breakfast frequency, supper frequency, staple food composition, eggs, milk, soy products, coffee, and desserts (Table 2).

**Table 2:** Univariate analysis results.

Variable	*Z*	*P*
Age	6.51	0.001
Gender	−9.45	0.001
BMI	17.75	0.001
WHR	21.17	0.001
WHtR	22.11	0.001
Sleep duration	−9.36	0.001
Exercise duration	−4.41	0.001
Frequency of breakfast	−4.80	0.001
Frequency of late-night snacks	8.38	0.001
Staple food composition	−2.75	0.006
Frequency of milk	−7.71	0.001
Frequency of egg	−11.10	0.001
Frequency of soy products	−5.02	0.001
Frequency of coffee	−10.56	0.001
Frequency of dessert	10.45	0.001

BMI, body mass index; WHR, waist-to-hip ratio; WHtR, waist-to-height ratio.

### Variance inflation factor

To improve the stability of the prediction model, a multicollinearity test was performed for statistically significant variables in univariate analysis. The variance inflation factor (VIF) was used to evaluate the degree of collinearity between variables, and VIF > 5 was set as the criterion for the existence of moderate collinearity; such variables were eliminated. The VIF values of the variables such as night snack (VIF = 14.35), eggs (VIF = 6.43), milk (VIF = 16.54), bean products (VIF = 9.65), and coffee (VIF = 11.44) all exceeded the threshold, indicating that they had strong collinearity with other variables, so they were excluded from the final model.

### Multivariate analysis

In this study, MAFLD was used as the dependent variable, and variables with a VIF < 5 were included in the multivariate binary logistic regression analysis. The results showed that age, gender, BMI, WHR, WHtR, sleep time, and dessert intake frequency were statistically significantly associated with the occurrence of MAFLD (*P* < 0.05). Among them, age (OR = 1.62, 95% CI 1.36, 1.92), BMI (OR = 2.15, 95% CI 1.75, 2.64), WHR (OR = 2.10, 95% CI 1.64, 2.69), WHtR (OR = 4.01, 95% CI 3.07, 5.23), and dessert intake frequency (OR = 1.46, 95% CI 1.17, 1.81), suggesting that these factors are independent risk factors for the development of MAFLD. In contrast, the OR values for women (OR = 0.42, 95% CI 0.31, 0.57) and adequate sleep (OR = 0.71, 95% CI 0.59, 0.85) were less than 1, suggesting that female and adequate sleep time may be protective factors for NAFLD (Table 3).

**Table 3:** Results of multivariate analysis.

Characteristic	Z	*P*	OR (95% CI)
Age	5.47	0.001	1.62 (1.36∼1.92)
Gender	−5.50	0.001	0.42 (0.31∼0.57)
BMI	7.28	0.001	2.15 (1.75∼2.64)
WHR	5.94	0.001	2.10 (1.64∼2.69)
WHtR	10.23	0.001	4.01 (3.07∼5.23)
Sleep duration	−3.65	0.001	0.71 (0.59∼0.85)
Frequency of dessert	3.41	0.001	1.46 (1.17∼1.81)
Exercise duration	1.19	0.24	1.11 (0.94∼1.31)
Frequency of breakfast	−1.02	0.31	0.92 (0.79∼1.08)
Staple food composition	−0.67	0.50	0.89 (0.63∼1.25)

OR, odds ratio; 95% CI, 95% confidence interval; BMI, body mass index; WHR, waist-to-hip ratio; WHtR, waist-to-height ratio.

### Interactive effect

Following multivariable screening of seven variables, interaction analyses were performed to assess synergistic or antagonistic effects on MAFLD. Significant interactions (*P* < 0.05) were observed between (1) age and BMI (OR = 1.68, 95% CI 1.35–2.10; *P* < 0.001), (2) WHR and sleep duration (OR = 1.28, 95% CI 1.05–1.56; *P* = 0.01), (3) WHtR and sleep duration (OR = 1.28, 95% CI 1.05–1.58; *P* = 0.02), (4) frequency of dessert intake and sleep duration (OR = 0.73, 95% CI 0.61–0.88; *P* < 0.001), (5) WHR and frequency of dessert intake (OR = 1.86, 95% CI 1.44–2.40; *P* < 0.001), and (6) WHtR and frequency of dessert intake (OR = 1.90, 95% CI 1.45–2.48; *P* < 0.001). No significant interactions were found for gender-BMI (*P* = 0.17), sleep duration-BMI (*P* = 0.77), frequency of dessert intake-BMI (*P* = 0.59), age -gender (*P* = 0.88), WHR-gender (*P* = 0.85), or WHtR-gender (*P* = 0.09) (Table 4).

**Table 4:** Statistical interactions between variables.

Characteristic	*β*	OR (95% CI)	*P*
Age * BMI	0.52	1.68 (1.35, 2.10)	<0.001
Gender * BMI	−0.25		0.17
Sleep * BMI	0.03		0.77
Dessert * BMI	0.07		0.59
Age * gender	−0.02		0.88
WHR * gender	0.04		0.85
WHtR * gender	−1.00		0.09
WHR * Sleep	0.25	1.28 (1.05, 1.56)	0.01
WHtR * Sleep	0.25	1.28 (1.05, 1.58)	0.02
dessert * Sleep	−0.31	0.73 (0.61, 0.88)	0.001
WHR * Dessert	0.62	1.86 (1.44, 2.40)	<0.001
WHtR * Dessert	0.64	1.90 (1.45, 2.48)	<0.001

*β*, regression coefficient; OR, odds ratio; 95% CI, 95% confidence interval; BMI, body mass index; WHR, waist-to-hip ratio; WHtR, waist-to-height ratio.

### Health index

**Figure 4:**
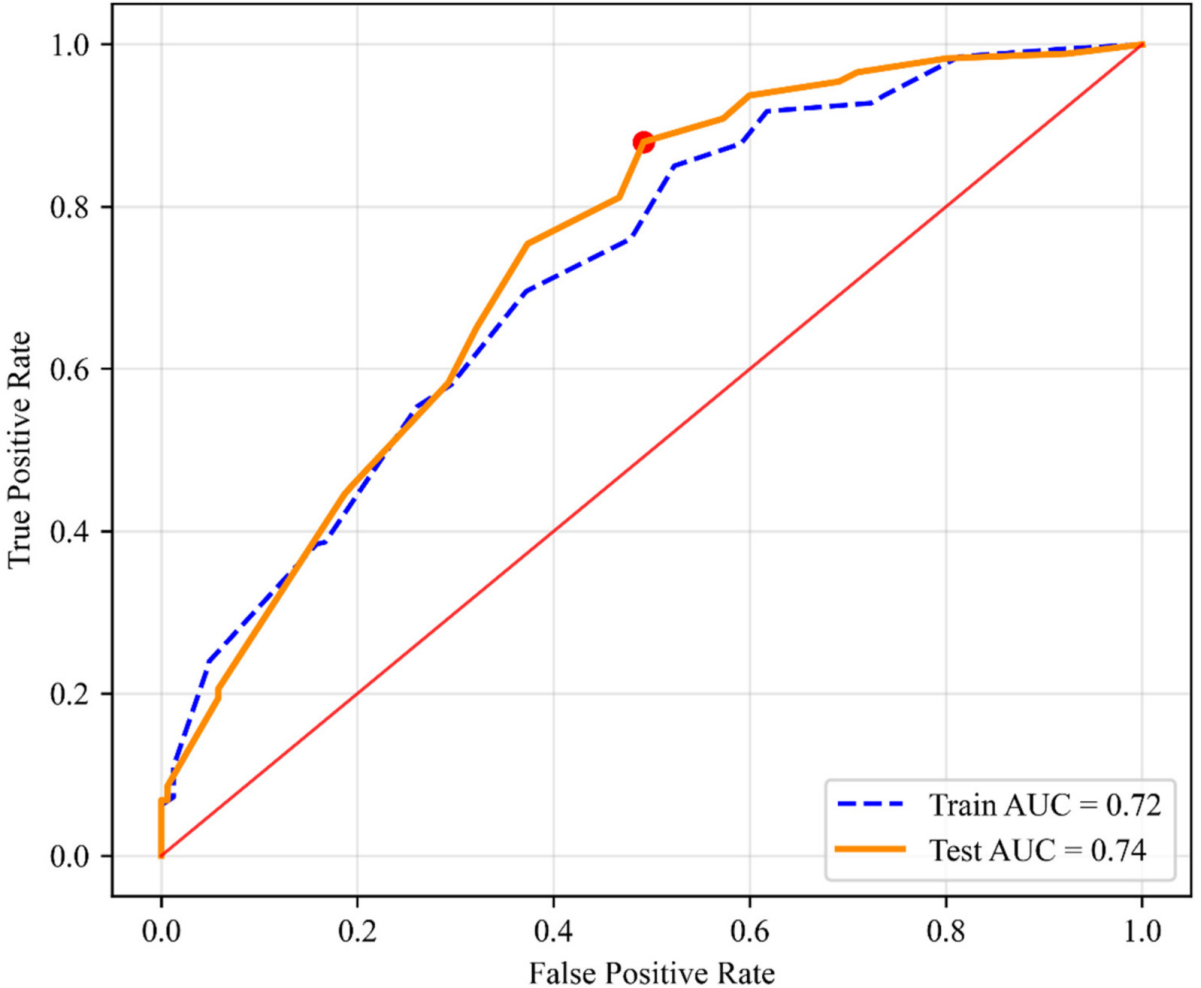
ROC curve of the health index predicts MAFLD risk.

Based on significant predictors identified by multivariable logistic regression analysis, a health index scoring system was developed (Table 5). The scoring criteria and weights for each variable were derived from standardized *β* coefficients (*P* < 0.05). Age (total 2 points), <14 years (2 points), ≥14 years (1 point); Gender (total 3.5 points), Female (3.5 points), Male (1.75 points); BMI (total 3 points), <18.9 (3 points), 18.9–21.5 (2 points), ≥21.5 (1 point); WHtR (total 5.5 points; strongest predictor), <0.46 (5.5 points), ≥0.46 (2.75 points); WHR (total 3 points), <0.85 (3 points), ≥0.85 (1.5 points); Sleep Duration (total 1.5 points), >8 h (1.5 points), 7–8 h (1.125 points), 6–7 h (0.75 points), <6 h (0.375 points); Dessert Intake (total 1.5 points; non-linear association), ≥6 times/week (0.375 points), 4–5 times/week (0.75 points), 2–3 times/week (1.125 points), ≤1 time/week (1.5 points). The theoretical range of the health index score is 8.75–20. ROC curve analysis determined an optimal diagnostic threshold of 11.5 (Youden index = 0.68). The model demonstrated good discrimination in both the training set (AUC = 0.72; 95% CI 0.68, 0.76, *P* < 0.001) and validation set (AUC = 0.74; 95% CI 0.70, 0.78, *P* < 0.001) (Figure 4). The Hosmer-Lemeshow test indicated good model calibration (*P* = 0.12). Predictor weights, ranked by standardized *β* coefficient magnitude, were WHtR (*β* = 1.39), gender (*β* = −0.86), BMI (*β* = 0.77), WHR (*β* = 0.74), ethnicity (*β* = 0.56), age (*β* = 0.48), dessert intake (*β* = 0.38), and sleep duration (*β* = −0.34). The assigned scores for each dimension showed high consistency with the direction and magnitude of the regression coefficients (Pearson's *r* = 0.93, *P* < 0.01).

**Table 5:** Health index.

Variable	Point
Age	
<14	2
≥14	1
Gender	
Male	1.75
Female	3.5
BMI	
<18.9	3
≥18.9, <215	2
≥21.5	1
WHtR	
<0.46	5.5
≥0.46	2.75
WHR	
<0.85	3
≥0.85	1.5
Sleep duration	
<6	0.375
≥6, <7	0.75
≥7, <8	1.125
≥8	1.5
Frequency of dessert	
≤1	1.5
≥2, <4	1.125
≥4, <6	0.75
≥6	0.375
Range	8.75–20

BMI, body mass index; WHR, waist-to-hip ratio; WHtR, waist-to-height ratio.

## Discussion

MAFLD has become a health problem that cannot be ignored in children, and it is gradually decreasing (27). This study analyzed the risk factors of MAFLD in children and confirmed the independent risk factors of anthropometric indicators (BMI, WHR, WHtR) and metabolic risk behaviors (lack of sleep, high-sugar diet) (17). Among them, the weight of WHtR (*β* = 1.39) was much higher than BMI (0.77). This finding is consistent with Li, C. et al., the abdominal obesity index can better predict NAFLD in the Chinese population, suggesting that in the child population, the abdominal obesity index can better reflect the metabolic harm of visceral fat ectopic deposition than BMI (28). This is related to the unique anatomical location and metabolic activity of visceral fat, which is usually directly diverted from visceral adipose tissue (such as mesentery and omentum fat) into the portal venous system, and the free fatty acids and adipokines released by it reach the liver directly without systemic dilution (29). In addition, the concentration of free fatty acids in the portal blood of visceral obese patients was much higher than that of systemic obese patients, which directly promoted the synthesis of fatty acids in hepatocytes and significantly increased the rate of liver TG synthesis (30). This study found that the detection rate of MAFLD gradually increased with age, which was consistent with the results of most investigators (31). This may be because as children age, sedentary time increases, and their diet deteriorates (high-sugar, high-fat foods), resulting in excess energy, visceral fat and liver fat deposits gradually increasing, and increased levels of sex hormones during puberty may affect fat distribution and metabolism, promoting visceral fat accumulation (32). Like adults, the detection rate of MAFLD in men is still higher than that in women, which is mainly related to the differences in sex hormones and fat distribution characteristics. At the same time, higher testosterone levels in men may promote visceral fat accumulation and increase liver fat accumulation (32, 33). This study found that the intake of high-frequency desserts will increase the detection rate of MAFLD, which may be because since common added sugars in desserts (such as sucrose and fructose syrup) contain a large amount of fructose, fructose, unlike glucose, its metabolism is not regulated by insulin, and it is mainly completed through the liver, and fructose is rapidly converted into triglycerides in the liver, resulting in fat accumulation in hepatocytes (34). At the same time, this study found that adequate sleep is a protective factor for MAFLD, and adequate sleep can reduce the risk of MAFLD by regulating hormones, inhibiting inflammation, and maintaining the balance of the biological clock (35). Research by Romero-Gómez, M. et al. demonstrated that physical exercise effectively reduces hepatic fat content (36). While this study also examined the relationship between exercise and fatty liver disease, no statistically significant association was observed. Subsequent telephone follow-up of 100 participants revealed that their reported exercise duration primarily reflected scheduled physical education (PE) class time. Further investigation, however, indicated that approximately 70% of students engaged in only minutes of actual active exercise during PE sessions—often exercising briefly followed by extended rest periods—resulting in significantly lower effective exercise duration than reported. Consequently, based on potentially overestimated exercise duration data, this study failed to establish a significant association between physical activity and fatty liver disease. Furthermore, research by Ioannou, G. N. et al. identified diabetes and insulin resistance as significant risk factors for MAFLD, mediated through disruption of lipid metabolism and induction of metabolic dysregulation (22). However, as the study cohort consisted exclusively of school-aged children and adolescents—a population with inherently low prevalence rates of diabetes and insulin resistance—no statistically significant association between these metabolic factors and fatty liver disease was observed in this cohort. Studies by Xuan, Y. et al. demonstrated that in patients younger than 20 years, the ROC for predicting MAFLD was 0.67 using triglyceride (TG), and 0.66 using aspartate aminotransferase (AST) (37). Research by Li, H. et al. confirmed an AUC of 0.61 for the triglyceride-glucose (TyG) index and 0.66 for the TyG index combined with TyG-BMI in predicting MAFLD (38). Compared to other predictive models, the health index developed in our study achieved AUC of 0.72 in the training cohort and 0.74 in the validation cohort for identifying individuals at high risk of MAFLD, surpassing the predictive performance of the aforementioned individual biomarkers as well as the TyG-BMI combination. Furthermore, this index is non-invasive and easily implementable.

In developing a predictive model for MAFLD risk, the dataset was randomly partitioned into training (70%) and validation (30%) sets. Notably, the model demonstrated marginally superior discriminative performance on the validation set, with an AUC of 0.74, compared to 0.72 on the training set. This observed discrepancy, where validation AUC exceeded training AUC, may be attributable to random sampling variability, potentially resulting in the validation cohort providing a slightly more representative characterization of underlying MAFLD risk factors (39, 40). Comparative analysis of key parameters revealed minimal differences between cohorts. Continuous variables (mean ± standard deviation) were comparable: Age (training: 12.78 ± 3.56 years vs. validation: 12.69 ± 3.56 years), BMI (19.94 ± 3.88 kg/m^2^ vs. 19.91 ± 3.76 kg/m^2^), WHR (0.83 ± 0.07 vs. 0.83 ± 0.07), and WHtR (0.46 ± 0.06 vs. 0.46 ± 0.06). Similarly, categorical variables showed no significant differences in the proportion of individuals with sleep duration <6 h (11.8% vs. 12.2%) or dessert consumption frequency ≥6 times/week (2.6% vs. 3.2%), although male gender distribution reached statistical significance (45.3% vs. 50.2%, *P* < 0.05). Overall, the minor variations between sets fall within expected ranges of random fluctuation. These results support the robust generalization capability of the developed health index prediction model.

The health index scoring system constructed in this study achieves methodological breakthroughs in multiple dimensions (18, 19). (1) The introduction of WHtR instead of the traditional waist circumference index is more suitable for the body shape changes of children during the growth spurt period; (2) By quantifying the effect of dessert intake frequency and sleep deprivation, the explanatory dimension of the traditional metabolic model was expanded. The stable prediction performance of the model in the validation set (AUC = 0.74) suggests that it can be used as a community-based primary screening tool for children's MAFLD, and can detect high-risk groups of MAFLD in time.

In school, community, or household settings, utilizing only a weighing scale and a measuring tape following standardized protocols enables the efficient collection of children's height, weight, waist circumference, and hip circumference. These measurements are then used to calculate BMI, WHR, and WHtR using established formulas. Integrating this anthropometric data with an assessment of children's sleep patterns and dietary habits allows for the assignment of scores to each indicator according to the health index scoring system proposed in this study. Aggregating these scores yields a comprehensive health index. This health index, based on the optimal cut-off value of 11.5 points, serves as a practical tool for the timely identification of children at high risk for MAFLD. The health index can enable high-risk children to obtain early intervention opportunities before liver enzyme abnormalities occur. Compared with the general screening scheme, the risk stratification model can reduce ineffective screening and meet the evaluation criteria of health economics (41). Especially in areas with scarce medical resources, the tool can be implemented through the school medical system to realize the closed-loop management of the school-family-community three-level prevention and control network.

The management of MAFLD necessitates multidisciplinary collaboration, focusing on reducing body weight and waist circumference, improving insulin resistance, preventing/treating T2DM, alleviating MASH, and reversing fibrosis (20, 25). All patients require health education for lifestyle modification, with pharmacological intervention indicated for those with metabolic cardiovascular risks or liver injury. Combined diet-exercise therapy is fundamental: Weight loss magnitude correlates positively with metabolic benefits in overweight/obese patients (3%–5% reduction reverses steatosis within 1 year; 7%–10% alleviates MASH; ≥ 10% reverses fibrosis; ≥15% may ameliorate T2DM) (25). A daily caloric deficit of 500–1,000 kcal achieves progressive weight loss and hepatic fat reduction, with efficacy demonstrated for low-carbohydrate, low-fat, intermittent fasting, and Mediterranean diets. For exercise, ≥150 min/week of moderate aerobic activity (e.g., brisk walking) or 3–5 HIIT sessions weekly reduces hepatic fat, improves cardiorespiratory fitness, and decreases waist circumference (showing a dose-response relationship) (25). Resistance training alone is reserved for patients with poor cardiorespiratory function. Crucially, combined diet-exercise therapy outperforms either modality in isolation and requires sustained personalized implementation (20, 25).

There are the following limitations in this study. (1) As the study cohort excluded children with diabetes and insulin resistance, the established relationship between these conditions and MAFLD could not be confirmed. Consequently, the predictive health index model developed herein does not incorporate diabetes or insulin resistance metrics. Therefore, the application of the 11.5 diagnostic threshold to children with insulin resistance or diabetes may fail to accurately identify their risk of developing MAFLD. (2) Dietary habits, primarily reported by guardians, may underestimate the frequency of sugary food intake, as guardians are often unaware of their children's dietary choices during school hours. (3) MAFLD diagnosis in this study relied on conventional ultrasonography performed with portable devices. The diagnostic accuracy of this method is inherently lower than that achieved with MRI-PDFF quantification. (4) The health index assessment system was developed and validated exclusively in a pediatric population. Thus, its applicability and performance in adult populations require further investigation.

Subsequent research will focus on: (1) developing a novel health index model incorporating insulin resistance and metabolic syndrome indicators for children with diabetes and insulin resistance, based on the current study, to establish diagnostic thresholds for identifying high-risk MAFLD individuals within this population; (2) constructing and validating a health index diagnostic model tailored for high-risk MAFLD adults and determining its optimal diagnostic threshold; (3) evaluating the effectiveness of implementing dietary control, lifestyle modification, and increased physical activity interventions for improving MAFLD in pediatric patients with a confirmed diagnosis.

The comprehensive health index scoring system provides a quantifiable decision-making tool for the precise prevention and control of MAFLD in children, and its standardized and low-cost characteristics meet the needs of the hierarchical diagnosis and treatment system. In the future, it is necessary to improve the adaptability of the model through multi-center verification and explore a dynamic risk early warning system based on artificial intelligence, to finally realize the paradigm shift from disease diagnosis to health risk management.

## Conclusion

This study showed that the occurrence of MAFLD was significantly independently associated with age, gender, ethnicity, BMI, WHR, WHtR, sleep duration, and high-frequency dessert intake (*P* < 0.05). The health index scoring system based on multi-dimensional risk factors showed good risk stratification ability, with the optimal prediction threshold of 12.25 (Youden index = 0.68), and the AUC of the training set and the validation set were 0.72 (95% CI 0.68–0.76) and 0.74 (95% CI 0.70–0.78), respectively. The model was well calibrated (Hosmer-Lemeshow *χ*^2^ = 7.21, *P* = 0.12), suggesting that it could be used as an early screening tool for high-risk groups of MAFLD in children and provide a quantitative basis for targeted intervention.

## Data Availability

The raw data supporting the conclusions of this article will be made available by the authors, without undue reservation.
